# Literature Review of Venous Spasm During Pacemaker Implantation

**DOI:** 10.7759/cureus.88682

**Published:** 2025-07-24

**Authors:** Juwayria A Ahmed, Khudheeja A Ahmed, Wasifuddin Syed, Mohammed Habeeb Ahmed

**Affiliations:** 1 Department of Research, Kaaj Healthcare, San Jose, USA; 2 College of Osteopathic Medicine, Touro University California, Vallejo, USA; 3 College of Medicine, Edward Via College of Osteopathic Medicine, Monroe, USA; 4 Department of Cardiology, Kaaj Healthcare, San Jose, USA

**Keywords:** axillary vein, cephalic vein, pacemaker, subclavian vein, venous spasm

## Abstract

Venous spasm is a rare yet clinically relevant complication during pacemaker implantation that can obstruct venous access and delay procedures. Despite its significance, it is infrequently reported in the literature and not clearly understood, thus lacking a standardized management protocol. This review aimed to synthesize current literature on venous spasm occurring during pacemaker or any other cardiac device implantation, highlight common features, and summarize effective management strategies. A comprehensive search of PubMed, Embase, and ScienceDirect through July 2025 identified 17 relevant sources, including five studies and 12 case reports. Axillary and subclavian venous spasm was most frequently reported, particularly in older female patients, and typically diagnosed by contrast venography following unsuccessful guidewire passage. Cephalic venous spasm was also noted infrequently. Management strategies varied, with reported interventions including temporary cessation of manipulation, use of hydrophilic guidewires, administration of intravenous nitroglycerin, adjustment of access puncture site, or contralateral venous access. In some cases, venous spasm was refractory to treatment, requiring procedural modification or abandonment. This review consolidates previously used successful management strategies into a proposed stepwise workflow and underscores the need for further research to improve understanding of venous spasm and to develop standardized treatment guidelines.

## Introduction and background

Cephalic vein (CV) cutdown and axillary/subclavian (AV/SV) vein puncture are the two primary methods for lead placement during pacemaker implantation, with the latter being the preferred method [1]. Venous spasm of the axillary or subclavian vein during pacemaker implantation is an infrequently reported but significant complication of SV/AV puncture, potentially resulting in failed access [2]. Venous spasm is generally defined as narrowing of the vessel lumen and is diagnosed via contrast venography [3]. Duan et al. classified venous spasm based on the degree of the lumen’s narrowing, categorizing mild venous spasm and severe venous spasm as a reduction in lumen caliber of 50%-90% and ≥ 90%, respectively [3,4].

The exact etiology of venous spasm remains unclear, though it is believed to represent a reflexive phenomenon in response to injury of surrounding tissues or venous walls during attempted puncture or due to increased catecholamine levels due to vein injury during vein puncture or caused by mechanical irritation of lead insertion or otherwise chemical irritation of the contrast agents [1,2,5]. One randomized study by Vitali et al. involving 270 patients found that in the event of unintentional arterial puncture following axillary vein cannulation using fluoroscopy, vasospasm of the vein may occur, complicating entry [6]. Additionally, according to some researchers, it has been proposed that the higher concentration of myocytes within the tunica media, the smooth muscle layer in the vascular wall responsible for both vasoconstriction and vasodilation, predisposes the AV and SV to contraction over relaxation [1]. Due to a scarcity of research on the topic, there is no standard treatment to manage venous spasm during pacemaker implantation [1,2]. Through this literature review, we aim to address this gap by compiling the known literature of venous spasm during pacemaker implantation and exploring the strategies utilized to successfully manage this complication.

## Review

Methods

We did a thorough search of PubMed, Embase, and ScienceDirect from inception through July 2025 (Table 1). We searched PubMed using the keywords and found 16 results, of which we kept 13 based on some exclusion criteria (Table 1). We searched Embase using the keywords and found 17 results, of which we kept 15 based on some exclusion criteria (Table 1). We searched ScienceDirect using the keywords and found 44 results, of which three were kept based on some exclusion criteria (Table 1). Our exclusion criteria included such aspects as lack of relevance to the subject matter and articles written in a language other than English (Table 1). Our inclusion criteria included aspects such as focused relevance to our subject matter of venous spasm during pacemaker implantation and articles written in the English language (Table 1). We found five studies and 12 case reports on the topic, with a total of 17 sources. 

**Table 1:** Literature review search databases and results.

Database	Search Entry	Results
PubMed	(venospasm OR "venous spasm" OR "vein spasm" NOT hemifacial) AND (pacemaker OR "cardiac electronic device implantation" OR "cardiac implantable electronic device implantation" OR "cephalic vein")	16 results, of which 13 were relevant to venous spasm during pacemaker implantation
Embase	(venospasm OR “venous spasm” OR “vein spasm” NOT hemifacial) AND (pacemaker OR “cardiac electronic device implantation”)	17 results, of which 2 were duplicates and 1 was irrelevant, leaving 15 results that were relevant to venous spasm during pacemaker implantation
ScienceDirect	(venospasm OR "venous spasm" OR "vein spasm" NOT hemifacial) AND (pacemaker OR "cardiac electronic device implantation")	44 results, of which 3 was relevant to venous spasm during pacemaker implantation

Studies of axillary venous spasm

There have been four studies done on the topic of venous spasm during pacemaker implantation, all of which related specifically to axillary venous spasm. A study by Steckiewicz et al. involving 403 patients found 12 cases of axillary vein spasm “(mean age 57 ± 25 years)” during cardiac implantable electronic device implantation [1]. Cases in which spasm caused failed AV puncture were managed successfully by switching to SV puncture [1]. No cases of SV spasms following SV puncture were found, which the authors attributed to the two adjacent bony structures maintaining the patency of the SV lumen [1]. 

A study by Duan et al. involving 40 patients undergoing contrast-guided AV puncture for implantation of a pacemaker or a defibrillator showed that patients who were given 200 µg nitroglycerin via the ipsilateral peripheral vein three minutes prior to vein puncture showed a decreased occurrence of “mild or severe venous spasm” [3,7]. Mild venous spasm, characterized as a decrease in luminal caliber of 50-90%, was found with an incidence of 35% and 10% in the control group and nitroglycerin group, respectively [7]. Severe venous spasm, characterized by a decrease in luminal caliber of ≥ 90%, was found with an incidence of 20% and 5% in the control group and nitroglycerin group, respectively [7]. Severe venous spasm resulted in failed puncture for three minutes in three patients, after which SV puncture was used instead [7]. 

A prospective study on venous spasms, conducted by Duan et al., involved 74 patients undergoing pacemaker or defibrillator implantation with fluoroscopy and contrast-guided AV access [8]. The study found that AV spasm may be more common than initially expected, finding 22 patients (29.7%) exhibiting mild venous spasm and six patients (8.1%) exhibiting severe venous spasm, as defined during the prior study [8]. The highest occurrence of severe venous spasm was found among females over 70 years of age [8]. Moreover, this phenomenon was observed to exhibit an independent negative correlation with both the overall success rate and the success rate within a three-minute timeframe [8]. Again, all three patients with failed access also exhibited severe venous spasm, which was managed by changing the access site to the SV instead [8].

A recent cross-sectional study by al-Gburi involving 137 patients undergoing permanent pacemaker or implantable cardioverter-defibrillator implantation via AV puncture found similar results [5]. Grade I and Grade II AV spasm were defined as a 50-90% and >90% luminal diameter reduction, respectively. Grade I AV spasm occurred in 38 patients (27.8%), whereas Grade II AV spasm was documented in eight patients (5.8%) [5]. Patients exhibiting AV spasm showed significantly decreased rates of successful puncture within five minutes [5]. Additionally, patients exhibiting AV spasm were more likely to be females over the age of 70 years [5]. 

Case reports of AV spasm

We found seven case reports of AV spasm during pacemaker implantation. 

A report by Cooper et al. detailed the case of an 83-year-old female who presented with syncope and complete heart block [9]. AV access was initially attempted on the right side due to recent surgery in the left shoulder. However, efforts to navigate both standard and hydrophilic guidewires ended repeatedly in failure, and the procedure was abandoned due to patient discomfort [9]. The following day, access was attempted again, this time on the left side. Initial venography before the procedure showed veins of reasonable size. However, attempts to advance a guidewire were once again unsuccessful, and repeated venography revealed significant narrowing of the vein [9]. However, after some time, the vein relaxed, and a dual-chamber pacemaker was successfully placed via a hydrophilic wire [9]. 

A report by Hiruma et al. documented a novel case of an 83-year-old male with a history of complete atrioventricular block undergoing dual-chamber pacemaker implantation via left AV puncture [10]. Initial venography showed normal vein anatomy, and initial puncture demonstrated appropriate backflow, but the guidewire was unable to gain entry [10]. Repeated venography showed disappearance of the left axillary vein “on the first limb” and enlarged collaterals [10]. Fifteen minutes after intravenous administration of 1000 μg of nitroglycerine, the spasm had not resolved, so right AV access was attempted [10]. Once again, venography showed complete occlusion of the axillary vein, and severe venous spasm was suspected [10]. Since the left CV was visible, the bailout technique used in this case was a partial cut-down of the left CV [10]. Due to the narrowness of the CV, the leads were not directly inserted into the vein but instead were inserted through a single inserter through which the guidewires were successfully advanced [10]. After this, two sheaths were successfully placed into the cephalic vein [10]. The ventricular lead was implanted through a sheath in the right ventricular septum, while the atrial lead was implanted through a sheath in the right atrial appendage [10].

A case report by Chan et al. discussed the report of a 74-year-old woman with paroxysmal atrial fibrillation and common sinus pauses of a maximum of 5 seconds undergoing a dual-chamber pacemaker implantation via AV puncture [11]. Although the first AV puncture was successful, the second puncture was repeatedly unsuccessful [11]. Venography revealed venous spasm of the axillary vein from the rib cage’s lateral edge to the extrathoracic region of the SV [11]. The spasm persisted despite administration of 200 and 400 µg of nitroglycerin [11]. Finally, the puncture of the AV lateral to the spasm was successful, through which the guidewire was inserted [11]. Even about an hour later, the spasm was still visible on venography [11]. A clinical vignette by Bhasin et al. presented a case of a woman in her 60s with a history of complete heart block undergoing pacemaker implantation via left AV puncture [12]. After unsuccessful attempts to access the vein, venography was repeated and showed severe spasm [12]. The spasm did not respond to normal saline infusion via ipsilateral peripheral venous access but resolved 10 minutes after administration of intravenous nitroglycerin, after which the vein was successfully accessed [12].

A clinical vignette by Duan et al. presented the case of a 75-year-old female with atrial fibrillation and complete atrioventricular block undergoing pacemaker implantation via left AV puncture [13]. Although initial venography showed normal vein anatomy, attempts to puncture the vein did not result in good venous return, and the guidewire could not be advanced [13]. Repeated venography showed severe venous spasm [13]. The spasm did not respond to nitroglycerine and did not relieve for over half an hour, after which a single-chamber pacemaker was implanted by changing the access site to the right axillary vein [13].

A clinical vignette by Maffe et al. describes a 70-year-old individual with sick sinus syndrome who was admitted for the placement of a dual-chamber pacemaker [14]. The patient had a prior history of atrial fibrillation, for which ablation had been attempted without success, leaving them symptomatic with varying episodes of bradycardia [14]. Imaging showed the axillary vein to be structurally sound and of adequate size [14]. However, when access was attempted, entry of the guide wire was unsuccessful [14]. Imaging through ultrasound and venography showed a significant decrease in vessel diameter, verified as venospasm [14]. Due to hypotension, no vasodilators were prescribed [14]. Fortunately, the intrathoracic segment of the subclavian vein maintained normal diameter, allowing the surgical team to successfully place the implant after switching to subclavian entry [14]. Follow-up imaging done hours after demonstrated resolution of the axillary vein spasm and a return to standard vessel caliber [14]. 

We found a related case of a 55-year-old male exhibiting AV spasm during cardiac device implantation [15]. The spasm did not respond to nitroglycerin [15]. A 0.014 hydrophilic guidewire was successfully sent through the median left cubital vein, crossing the spastic segment, and a 2.5 x 20 mm balloon was used to dilate the occlusion site, after which some blood flow returned and puncture was successfully performed distal to the balloon marker [15].

Case reports of SV spasm**

We found two case reports of SV spasm during pacemaker implantation. 

A report by Krishnappa et al. described the case of a 45-year-old woman who went through left extrathoracic subclavian vein puncture for pacemaker implantation [16]. Several unsuccessful attempts were made to advance the guidewire, after which the subclavian vein went into venous spasm [16]. Nitroglycerine was not administered due to a systolic blood pressure of 90 mmHg [16]. Since the pocket was already created, contralateral access was not preferable [16]. However, venography showed retrograde flow medially in the subclavian vein via collateral vessels, and medial subclavian vein puncture was successfully performed [16]. The authors of this report believed that a structurally prominent valve distally in the subclavian vein was responsible for impeding guidewire advancement, and the subsequent multiple attempts to advance the guidewire are what ultimately caused the venous spasm [16].

A clinical vignette by Koza et al. documented the report of a 60-year-old female with a past medical history of coronary artery bypass graft surgery and sinus pauses of up to 5 seconds undergoing dual-chamber pacemaker implantation via left subclavian vein puncture [17]. After multiple but unsuccessful attempts to cannulate the left SV, venography was performed, revealing SV spasm [17]. The spasm did not respond to incremental doses of nitroglycerin [17]. A lateral approach was attempted, which successfully cannulated the subclavian vein but without good venous return [17]. Finally, switching to a hydrophilic guidewire was successful [17].

Case reports of both AV and SV spasm

We found three case reports of severe spasm of both the AV and SV upon attempted access to the left AV. A case report by Vemuri et al. involved a 72-year-old female with complete heart block undergoing single-chamber permanent pacemaker implantation [2]. Initial venography showed no abnormalities in the anatomy of the AV and SV, but repeated attempts to cannulate the AV were unsuccessful [2]. Repeated venography showed severe spasm of both AV and SV [2]. The spasm did not respond to administration of 200 and 400 µg of nitroglycerin [2]. Instead, the right AV was cannulated successfully using anatomical landmarks and without venography to avoid venous spasm [2]. The authors of this case considered several potential causes for the venous spasm. Venous thrombosis was unlikely, considering the patient was on anticoagulants [2]. Local hematoma causing spasm through compression of the AV was also unlikely, considering the absence of contrast flow from as far back as the distal AV [2]. The physical impact of multiple punctures was also ruled out as a causative factor, considering the spasm occurred prior to the second access attempt [2]. The authors concluded that in this case, severe venous spasm of the AV and SV was likely a reaction to the contrast agent [2].

An oral presentation by Oktavio et al. documented the case of a 55-year-old female undergoing a dual-chamber pacemaker implantation via left AV puncture [18]. After repeated unsuccessful attempts to cannulate the vein, repeated venography indicated spasm of the AV and SV [18]. Fifteen minutes after the administration of a 10 mg intravenous bolus of isosorbide dinitrate, the spasm resolved partially, and a single-chamber pacemaker was placed instead [18]. 

A case report by Venet et al. noted the case report of a 66-year-old female undergoing a pacemaker implantation and atrioventricular node ablation to manage symptomatic atrial fibrillation and atypical atrial flutter [3]. After repeated attempts to access the axillary vein failed, venography revealed AV spasm [3]. SV access was attempted and similarly failed, after which venography revealed SV spasm [3]. The authors explained that since intravenous nitroglycerin is not available in France, isosorbide dinitrate was administered along with normal saline perfusion [3]. However, both the AV and SV spasms persisted after 15 minutes, and the procedure was terminated due to patient discomfort [3]. 

Study of cephalic vasoconstriction**

Cephalic venous (CV) spasm has been known to occur in the placement of totally implantable venous access ports (TIVAP), which are placed using central veins, similar to pacemaker implantation [19,20]. This complication has contributed to procedural failure in about 7.5% of cases [19,20]. One retrospective research study by Velez in the time range of 2008 and 2021 studied the effectiveness of cephalic vein cutdown (CVC) using ultrasound in the placement of 1,047 TIVAPs across 998 oncological cases [20]. CVC and subclavian vein puncture (SVP) were the preferred entry techniques in 82% and 18% of cases, respectively [20]. Venospasm was identified as the reason for procedural failure in 1.5% of patients since vessel diameters under 3.5 mm didn’t allow for successful catheter placement via CVC [20]. In these cases complicated by spasm, the procedure was completed using SVP as an alternative entry point [20]. The study further confirmed that failed CVC attempts may be converted to SVP through that initial surgical site, with a documented success rate of 99.8%, consistent with findings by Di Carlo and Toro [20]. 

We found one study, also by Steckiewicz et al., exploring cephalic vein vasoconstriction throughout cardiac implantable electronic device placement [19]. The study included 146 procedures involving cephalic vein cutdown and found vasoconstriction of the cephalic vein in 11 patients “(mean age 59.0 ± 21.2 years)” [19]. In three cases, guidewires were successfully passed through the constricted CV region [19]. In two cases, CV vasoconstriction spontaneously resolved, and successive attempts at lead advancement were successful [19]. Six of these cases were managed by switching to AV/SV puncture to place the leads [19]. 

Management approach

A compilation of the successful strategies utilized in the literature suggests a possible stepwise approach. Prophylactic treatment with nitroglycerin prior to the procedure has been associated with a reduced incidence of venous spasm. If the procedure results in venous spasm, the literature suggests a short period of procedural pause wherein clinicians allow several minutes for the spasm to resolve. If spasm persists, the use of a hydrophilic guidewire may facilitate vascular access. In cases requiring further intervention, incremental doses of intravenous nitroglycerin have been reported as appropriate hemodynamically. Additional imaging with contrast venography may assist with identifying alternative venous access points. In more resistant cases, contralateral venous access has been utilized successfully. These strategies consolidate the interventions that have proven successful in the literature, though further investigation is necessary to establish standardized guidelines.

## Conclusions

Venous spasm during pacemaker or other cardiac device implantation remains a rare but clinically significant complication that can result in failed vascular access and either procedural delay or termination. Although venous spasm is infrequently reported and lacks a standardized management protocol, our literature review provides further insight into the potential etiologies of venous spasm and consolidates previously used management strategies, as well as guidance to develop future management protocols. While various interventions have demonstrated success regarding intra-procedural venous spasm, the scope of the existing literature is limited. Therefore, further studies are needed to better understand the pathophysiology of venous spasm, identify consistent risk factors, and develop evidence-based guidelines for its management during pacemaker as well as other cardiac device implantation procedures.
